# Design, Synthesis and Anti-HIV Integrase Evaluation of *N*-(5-Chloro-8-Hydroxy-2-Styrylquinolin-7-yl)Benzenesulfonamide Derivatives

**DOI:** 10.3390/molecules15031903

**Published:** 2010-03-16

**Authors:** Zi-Guo Jiao, Hong-Qiu He, Cheng-Chu Zeng, Jian-Jun Tan, Li-Ming Hu, Cun-Xin Wang

**Affiliations:** College of Life Science & Bioengineering, Beijing University of Technology, Beijing, China; E-Mail: jiaoziguo@emails.bjut.edu.cn (Z.-G.J.)

**Keywords:** styrylquinoline derivatives, HIV-1 IN inhibitors, *N*-(styryl-8-hydroxyquinolin-7-yl)-benzenesulfonamide derivatives

## Abstract

Styrylquinoline derivatives are demonstrated to be HIV-1 integrase inhibitors. On the basis of our previous CoMFA analysis of a series of styrylquinoline derivatives, *N*-[(2-substituted-styryl)-5-chloro-8-hydroxyquinolin-7-yl]-benzenesulfonamide derivatives were designed and synthesized, and their possible HIV IN inhibitory activity was evaluated.

## Introduction 

**Figure 1:**
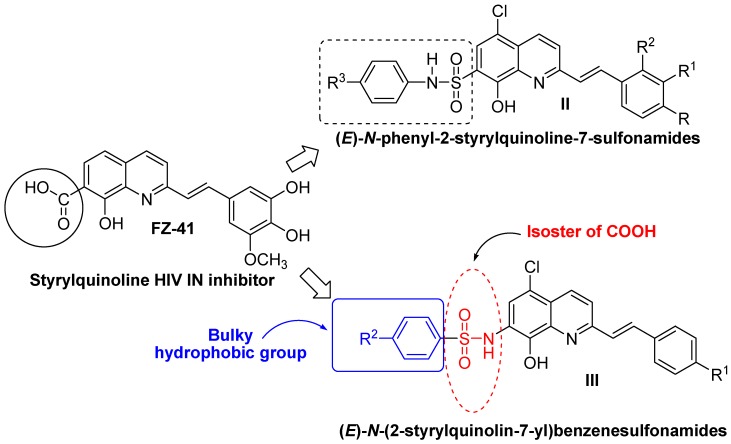
The design of *N*-(2-styrylquinolin-7-yl)benzenesulfonamides as potential HIV IN inhibitors.

There has been an increasing attention in the development of HIV integrase (IN) as a promising anti-HIV target, due to the fact that HIV IN is essential in the replication of HIV-1 and there are no similar enzymes involved in human cellular functions [1,2]. Therefore, extensive efforts have been made, resulting in a large number of HIV IN inhibitors [3,4], among which polyhydroxylated styrylquinolines have displayed an antiviral activity in a *de novo* infection assay of CEM4 cells, thereby opening an exciting structural platform for the design of new anti-HIV drugs [5,6,7,8]. The structure-activity-relationship of these compounds reveals that for *in vitro* activity a carboxyl group at C-7, a hydroxyl group at C-8 (salicylic acid structure) in the quinoline subunit and an ancillary phenyl ring are required. For example, FZ-41 in Figure 1 is one of the typical styrylquinoline-type HIV IN inhibitors [5,6,7,8]. 

To better understand the pharmacophore properties of styrylquinoline derivatives and to further design potential HIV-IN inhibitors, we recently investigated 38 styrylquinoline derivatives employing a comparative molecular field analysis (CoMFA) method [9]. The results indicated that inhibitory activity should be increased if a bulky group was near the carboxyl group at C-7 in the quinoline ring. Simultaneously, the presence of H-bonding donor is favorable near the C-7 atom, which might form a stable H-bond with some protein residues.

On the basis of the above information, we decided to modify the basic scaffold of styrylquinoline-type HIV IN inhibitors by replacing the carboxylic functionality at the C-7 position with an aromatic sulfonamide as its bioisosteric functionality, bearing a bulky aromatic group and, meanwhile, maintaining the feature of the H-bonding donor. In addition, to increase the acidic properties of the C-8 phenolic OH, a chloride atom was introduced at the C-5 of the quinoline ring. Such a design resulted in the target *N*-phenyl-2-styrylquinoline-7-sulfonamides **II** and *N*-(styrylquinolin-7-yl)-benzenesulfonamides **III** (Figure 1). Very recently, we have reported the synthesis and HIV IN screening of type **II** compounds [10]. In this work, we further describe the synthesis of type **III** compounds and their inhibitory activity against HIV IN. It should be pointed out that most of the work on the modification of styrylquinoline-type HIV IN concentrated on the modification of ancillary phenyl ring and linker unit. To the best of our knowledge, this is the first report concerning the replacement of salicyclic acid moiety of styrylquinoline HIV-IN inhibitors modified by sulfonamide.

## Results and Discussion 

### Synthesis of N-(2-styrylquinolin-7-yl)benzenesulfonamide derivatives

**Scheme 1:**
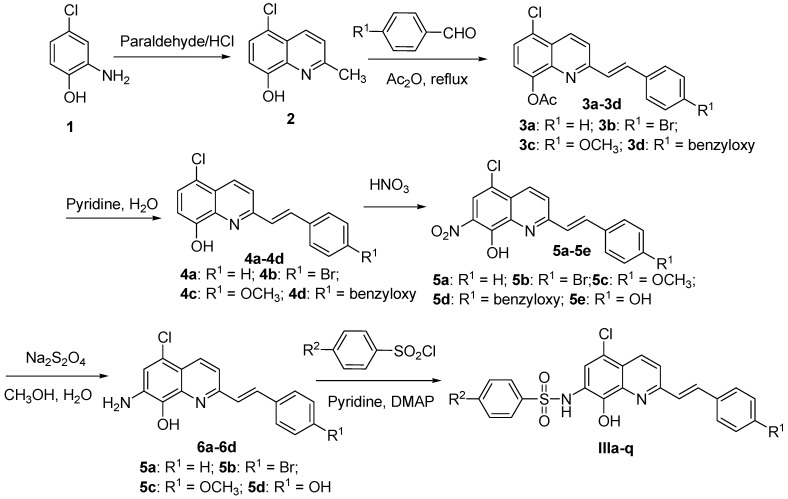
Synthesis of styrylquinolin-7-yl-benzenesulfonamide derivatives **III**.

The synthesis of styrylquinoline-type HIV IN is well-documented in the literature [5,6,7,8,11]. The typical procedures are involved in the Perkin condensation of 2-methylquinoline and aromatic aldehyde [5,6,7,8], or the Wittig reaction between triphenylphosphonium salts and various benzaldehydes under basic conditions [11]. Obviously, the Perkin reaction process is more convenient than the Wittig reaction. Consequently, the synthesis of designed *N*-(5-chloro-8-hydroxy-2-styrylquinolin-7-yl)benzenesulfonamide derivatives was carried out as shown in Scheme 1. The starting 5-chloro-quinolin-8-ol (**2**) was easily prepared from 2-amino-4-chlorophenol (**1**) according to the known procedure [16]. The Perkin condensation between **2** and various aromatic aldehydes generated 5-chloro-2-styrylquinolin-8-yl acetates **3** with pure *E* geometry, which were hydrolyzed in pyridine/water to give 5-chloro-2-styrylquinolin-8-ols **4**. After nitration and reduction, compounds **4** was converted to 2-styryl-7-amino-5-chloroquinolin-8-ols **6**. The title styrylquinolin-7-yl-benzenesulfonamide derivatives **III** were finally produced in 16-56% yield after reaction with benzenesulfonyl chloride derivatives (Scheme 1 and Table 1).

**Scheme 2:**
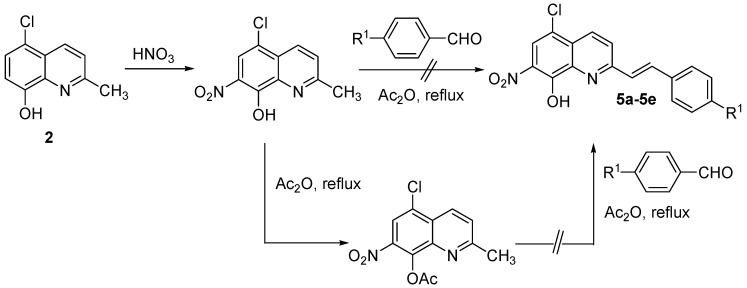
Reaction between 5-chloro-2-methyl-7-nitroquinolin-8-ol and aldehydes.

During the synthetic processes, the construction of the styryl scaffold by Perkin condensation reaction between 2-methylquinoline derivatives and various aldehydes was one of the key steps. According to a modified procedure [5,6], the 5-chloro-2-styrylquinolin-8-ols **4a-4d** were isolated in 32–57% yield by column chromatography. It is noteworthy that the smooth Perkin condensation is likely dependent on the nature of the 2-methylquinoline component. In an initial attemption to construct the styryl scaffold, we tried to carry out Perkin conditions with 5-chloro-2-methyl-7-nitro-quinolin-8-ol, but after refluxing in acetic anhydride for 7 days, no condensation product was detected and only 5-chloro-2-methyl-7-nitroquinolin-8-yl acetate was isolated (Scheme 2).

Selective sulfonylation reaction was also observed to be essential for the synthesis of title products **III**. In the presence of either pyridine or triethylamine, reaction of 2-styryl-7-amino-5-chloroquinolin-8-ols **6** and sulfonyl chloride always generated a mixture of *N*- and *O*-sulfonylation products, that proved to be hard to separate by conventional techniques. These results are quite different from the case of *o*-aminophenol, which was reported to give selective *N*-tosylation or *O*-tosylation by using 1 equiv. of pyridine or triethylamine, respectively [12]. Finally, we found that utilizing DMAP as a catalyst and pyridine as the solvent, the desired sulfonamide analogues could be synthesized smoothly [13].

The structures of styrylquinolin-7-yl-benzenesulfonamide derivatives **III** were confirmed by ^1^H-NMR, ^13^C-NMR, IR, and ESI-MS. Taking compound **IIIa** as an example, in its ^1^H-NMR spectrum, there are two AA’BB’ systems coupling in the range of 7.02 and 8.23 ppm, attributed to the proton signals of two benzene rings. The ethenyl linker shows two signals at 7.29 and 8.23 ppm (AB system) with a coupling constant of 16.0 Hz. This observation indicates that the styrylquinoline scaffold is in a *trans* configuration. In addition, the signals of three protons in quinoline ring (C-3, C-4 and C-6) exhibited a couple of doublets and a singlet at slightly low field at 7.75 (doublet), 8.34 (doublet) and 7.56 (singlet) ppm, respectively. The structure of **IIIa** (as well as for all compounds) was also characterized by ESI-MS. Strong peaks at 496.8 and 518.8 were recorded, which correspond to [M+H]^+^ and [M+Na]^+^.

### HIV IN inhibitory activity

All title compounds **IIIa-q** were preliminarily tested against purified HIV IN to determine any inhibitory activity possessed on the strand transfer reaction of IN. Using the high-throughput format assay approach developed by us [14], the inhibition percentages of styrylquinolin-7-yl-benzenesulfonamide derivatives **IIIa-q** were calculated based on the positive (baicalein) and negative (10% DMSO) controls and are listed in Table 2. For comparison, the IC_50_ data of baicalein and FZ-41 were also included. 

As shown in Table 2, compounds **IIIn-IIIq**, in which a free *para*-hydroxy group is present, showed higher inhibitory activity than that of the positive control, whereas, when the hydroxyl group was replaced by an electron-donating group (such as methoxy, compounds **IIIa-IIId**), hydrogen (**IIIe-IIIi**), or an electron-withdrawing group (such as bromide, compounds **IIIj-IIIm**), only a moderate inhibitory rate was observed. This observation means that the free hydroxyl moiety of styrylquinolin-7-yl-benzenesulfonamide derivatives is required for the inhibitory activity against HIV-IN. In addition, it was observed that the electron-withdrawing group at the *para*-position of benzenesulfonamide, such as nitro, may favor the inhibitory activity. For example, compound **IIIi** exhibits 96.7% inhibitory rate which decreases to 82.0% and 72.9% when the nitro was replaced by methyl (**IIIf**) and methoxy groups (**IIIg**), respectively. This results is not surprising because the electron-withdrawing group at the *para*-position of benzenesulfonamide moiety will increase the acidity of the benzenesulfonamide and lead to easier chelating with co-enzyme (mostly metallic ions), which is essential for the HIV IN inhibitory activity [15]. 

## Experimental

### General

All solvents were of commercial quality and were dried and purified by conventional methods. Melting points (mp) were determined on an XT4A Electrothermal apparatus equipped with a microscope and are uncorrected. Infrared spectra (IR) were recorded as thin films on KBr plates with a Bruker IR spectrophotometer and are expressed in *v* (cm^-1^). The ^1^H- and ^13^C-NMR spectra were obtained using an AV 400M Bruker spectrometer in CDCl_3_ or DMSO-*d_6_* with TMS as internal reference. The MS spectra (ESI) were recorded on a Bruker Esquire 6000 mass spectrometer. 

### General procedure for the Synthesis of (E)-5-Chloro-2-styryl-substituted Quinolin-8-ol Derivatives ***4***

A mixture of 5-chloro-2-methylquinolin-8-ol (**2**, 20 mmol) [16] and the appropriate benzaldehydes (60 mmol) in acetic anhydride (50 mL) was heated under reflux for 48 hrs and concentrated *in vacuo*. The black residue that formed was dissolved in a mixed solution of pyridine (40 mL) and water (10 mL), and the resulting solution was subjected to reflux for 3 hrs. After removal of pyridine, the formed solid was dissolved in CH_2_Cl_2_, washed three times by water, and dried over MgSO_4_. The solvent was removed under rotary evaporation. The desired compounds **4** were finally isolated by column chromatograph eluted using a mixture of petroleum ether and ethyl acetate. 

*(E)-5-Chloro-2-styrylquinolin-8-ol* (**4a**): Yield: 50%; mp: 146–147 ^o^C; ^1^H-NMR (400 MHz, CDCl_3_) *δ* 7.08 (d, 1H, *J* = 8.0 Hz, Ar-*H*), 7.33 (d, 1H, *J* = 16 Hz, -C*H*=C*H*-), 7.36 (t, 1H, *J* = 8.0 Hz, Ar-*H*), 7.43 (t, 2H, *J* = 8.0 Hz, Ar-*H*), 7.44 (d, 1H, *J* = 8.0 Hz, Ar-*H*), 7.64 (d, 2H, *J* = 7.2 Hz, Ar-*H*), 7.72 (d, 1H, *J* = 8.8 Hz, pyridine-*H*), 7.74 (d, 1H, *J* = 16.4 Hz, -CH=C*H*-), 8.44 (d, 1H, *J* = 8.8 Hz, pyridine-*H*); ^13^C-NMR (100 MHz, CDCl_3_) *δ* 110.3, 120.4, 121.0, 125.2, 126.9, 127.3, 127.4, 128.9, 129.1, 133.8, 135.4, 136.1, 151.1, 154.1; IR (KBr): *ν* 3,382, 3,022, 1,628, 1,500, 1,451, 1,251, 1,198 cm^-1^; ESI-MS: *m/z* 281.8 (M+H)^+^, 303.8 (M+Na)^+^.

*(E)-2-(4-Bromostyryl)-5-chloroquinolin-8-ol* (**4b**): Yield: 57%; mp: 195–196 ^o^C; ^1^H-NMR (400 MHz, CDCl_3_) *δ* 7.08 (d, 1H, *J* = 8.0 Hz, Ar-*H*), 7.30 (d, 1H, *J* = 16 Hz, -C*H*=C*H*-), 7.44 (d, 1H, *J* = 8.4 Hz, Ar-*H*), 7.48 (d, 2H, *J* = 8.4 Hz, Ar-*H*), 7.54 (d, 2H, *J* = 8.8 Hz, Ar-*H*), 7.65 (d, 1H, *J* = 16.4 Hz, -C*H*=C*H*-), 7.68 (d, 1H, *J* = 8.8 Hz, pyridine-*H*), 8.23 (s br, 1H, O*H*), 8.44 (d, 1H, *J* = 8.8 Hz, pyridine-*H*); IR (KBr): *ν* 3,434, 1,638, 1,586, 1,557, 1,497, 1,458, 1,392, 1,312, 1,172 cm^-1^; ESI-MS: *m/z* 359.6 (M+H)^+^, 357.6 (M^-^-H).

*(E)-2-(4-Methoxystyryl)-5-chloroquinolin-8-ol* (**4c**): Yield: 57%; mp: 195–196 ^o^C; ^1^H-NMR (400 MHz, CDCl_3_) *δ* 3.86 (s, 3H, OC*H*_3_), 6.94 (dt, 2H, *J* = 8.8 Hz, *J* = 2.0 Hz, Ar-*H*), 7.06 (d, 1H, *J* = 8.0 Hz, Ar-*H*), 7.20 (d, 1H, *J* = 16 Hz, -C*H*=C*H*-), 7.41 (d, 1H, *J* = 8.4 Hz, Ar-*H*), 7.57 (dt, 2H, *J* = 8.4 Hz, *J* = 2.0 Hz, Ar-*H*), 7.68 (d, 1H, *J* = 8.8 Hz, pyridine-*H*), 7.68 (d, 1H, *J* = 17.6 Hz, -C*H*=C*H*-), 8.41 (d, 1H, *J* = 8.8 Hz, pyridine-*H*); ^13^C-NMR (100 MHz, CDCl_3_) *δ* 55.4, 110.4, 114.4, 120.4, 121.0, 125.0, 125.3, 126.6, 128.8, 128.9, 133.5, 135.0, 138.4, 151.1, 154.6, 160.5; IR (KBr): *ν* 3,411, 3,031, 2,934, 2,841, 1,603, 1,513, 1,460, 1,152 cm^-1^; ESI-MS: *m/z* 312.0 (M+H)^+^, 309.7 (M^-^-H).

*(E)-2-(4-Benzyloxystyryl-5-chloroquinolin-8-ol* (**4d**): Yield: 32%;mp: 168–170 ^o^C; ^1^H-NMR (400 MHz, CDCl_3_) *δ* 5.12 (s, 2H, -C*H*_2_), 7.02 (d, 2H, *J* = 8.4 Hz, Ar-*H*), 7.07 (d, 1H, *J* = 8.4 Hz, Ar-*H*), 7.21 (d, 1H, *J* = 16.4 Hz, -C*H*=C*H*-), 7.36 (tt, 1H, *J* = 7.2 Hz, *J* = 2.4 Hz, Ar-*H*), 7.39-7.47 (m, 5H, Ar-*H*), 7.58 (d, 2H, *J* = 8.8 Hz, Ar-*H*), 7.69 (d, 1H, *J* = 8.8 Hz, pyridine-*H*), 7.70 (d, 1H, *J* = 15.6 Hz, -C*H*=C*H*-), 8.45 (d, 1H, *J* = 8.8 Hz, pyridine-*H*); IR (KBr): *ν* 3,370, 1,622, 1,601,1,508, 1,456, 1,307, 1,237, 1,169 cm^-1^; ESI-MS: *m/z* 387.9 (M+H)^+^ 385.7 (M^-^-H).

### General Procedure for the Synthesis of (E)-5-Chloro-7-nitro-2-styryl-substituted Quinolin-8-ol Derivatives ***5a-d***

To a three-necked flask (250 mL) charged with a mixed solution of nitric acid (50 mL, 65–68%) and water (50 mL) and cooled by ice-water bath was added the appropriate 2-styryl substituted quinolin-8-ol **4a-d** (10 mmol). The reaction solution was stirred for 12 hrs under ice-water cooling and then 24 hrs at room temperature. After addition of water (100 mL), precipitate formed which was filtered, washed by water for three times and dried to generate the desired 7-nitro-2-styrylquinolin-8-ols **5a-d**.

*(E)-5-Chloro-7-nitro-2-styrylquinolin-8-ol* (**5a**): Yield: 90%; mp: 170–172 ^o^C; ^1^H-NMR (400 MHz, DMSO-*d*_6_) *δ* 7.40 (t, 1H, *J* = 7.2 Hz, Ar-*H*), 7.48 (t, 2H, *J* = 7.2 Hz, Ar-*H*), 7.55 (d, 1H, *J* = 16 Hz, -C*H*=C*H*-), 7.73 (d, 2H, *J* = 7.6 Hz, Ar-*H*), 8.09 (d, 1H, *J* = 8.8 Hz, pyridine-*H*), 8.13 (s, 1H, Ar-*H*), 8.35 (d, 1H, *J* = 16 Hz, -C*H*=C*H*-), 8.53 (d, 1H, *J* = 8.8 Hz, pyridine-*H*), 11.66 (s, br, 1H, O*H*); ^13^C- NMR (100 MHz, DMSO-*d*_6_) *δ* 119.2, 121.2, 125.5, 126.7, 127.6, 128.0, 129.4, 129.7, 132.7, 134.1, 136.5, 137.7, 140.1, 150.0, 156.3; IR (KBr): *ν* 3,435, 1,568, 1,511, 1,328, 1,301, 1,252 cm^-1^; ESI-MS: *m/z* 324.5 (M^-^-H).

*(E)-2-(4-Bromostyryl)-5-chloro-7-nitroquinolin-8-ol* (**5b**): Yield: 89%; mp: 215–217 ^o^C; ^1^H-NMR (400 MHz, DMSO-*d*_6_) *δ* 7.58 (d, 1H, *J* = 16.4 Hz, -C*H*=C*H*-), 7.67 (s, 4H, Ar-*H*), 8.07 (d, 1H, *J* = 8.4 Hz, pyridine-*H*), 8.13 (s, 1H, Ar-*H*), 8.32 (d, 1H, *J* = 16.4 Hz, -C*H*=C*H*-), 8.54 (d, 1H, *J* = 8.4 Hz, pyridine-*H*), 11.66 (s, br, 1H, O*H*); ^13^C-NMR (100 MHz, DMSO-*d*_6_) *δ* 119.3, 121.3, 122.8, 125.6, 127.5, 127.6, 129.8, 132.4, 132.7, 134.2, 135.8, 136.3, 140.2, 150.0, 156.0; IR (KBr): *ν* 3,436, 1,631, 1,566, 1,518, 1,394, 1,344 cm^-1^; ESI-MS: *m/z* 404.5 (M^-^-H).

*(E)-2-(4-Methoxystyryl)-5-chloro-7-nitroquinolin-8-ol* (**5c**): Yield: 85%; mp: 154–156 ^o^C; ^1^H-NMR (400 MHz, DMSO-*d*_6_) *δ* 3.83 (s, 3H, OC*H*_3_), 7.04 (d, 2H, *J* = 8.4 Hz, Ar-*H*), 7.40 (d, 1H, *J* = 16 Hz, -C*H*=C*H*-), 7.68 (d, 2H, *J* = 8.0 Hz, Ar-*H*), 8.05 (d, 1H, *J* = 8.4 Hz, pyridine-*H*), 8.11 (s, 1H, Ar-*H*), 8.29 (d, 1H, *J* = 16 Hz, -C*H*=C*H*-), 8.50 (d, 1H, *J* = 8.4 Hz, pyridine-*H*), 11.48 (s, br, 1H, O*H*); IR (KBr): *ν* 3,436, 2,968, 1,595, 1,567, 1,514, 1,380, 1,256 cm^-1^; ESI-MS: *m/z* 354.7 (M^-^-H).

*(E)-2-(4-Benzyloxystyryl)-5-chloro-7-nitroquinolin-8-ol* (**5d**): Yield: 94%; mp: 207–208 ^o^C; ^1^H-NMR (400 MHz, DMSO-*d*_6_) *δ* 5.18 (s, 2H, -C*H*_2_-), 7.13 (d, 2H, *J* = 8.8 Hz, Ar-*H*), 7.36 (t, 1H, *J* = 8.8 Hz, Ar-*H*), 7.42 (t, 2H, *J* = 7.2 Hz, Ar-*H*), 7.42 (d, 1H, *J* = 15.2 Hz, -C*H*=C*H*-), 7.48 (d, 2H, *J* = 6.8 Hz, Ar-*H*), 7.69 (d, 2H, *J* = 8.4 Hz, Ar-*H*), 8.05 (d, 1H, *J* = 8.8 Hz, pyridine-*H*), 8.11 (s, 1H, Ar-*H*), 8.30 (d, 1H, *J* = 16 Hz, -C*H*=C*H*-), 8.51 (d, 1H, *J* = 8.8 Hz, pyridine-*H*), 11.66 (s, br, 1H, O*H*); IR (KBr): *ν* 3,433, 1,566, 1,513, 1,385, 1,330, 1,237, 1,176 cm^-1^; ESI-MS: *m/z* 430.8 (M^-^-H).

*(E)-2-(4-Hydroxystyryl)-5-chloro-7-nitroquinolin-8-ol* (**5e**): To a 1:1 mixture of HCl and acetic acid (30 mL) in a round-bottle flask (100 mL) was added **5d** (5 mmol). The mixture was heated under reflux for 3 hrs till the completion of the reaction. After removal of solvent under reduced pressure, the yellow powder was washed with water to give compound **5e**. ^1^H-NMR (400 MHz, DMSO-*d*_6_) *δ* 6.85 (d, 2H, *J* = 8.8 Hz, Ar-*H*), 7.22 (d, 1H, *J* = 16 Hz, -C*H*=C*H*-), 7.53 (d, 2H, *J* = 8.4 Hz, Ar-*H*), 7.70 (d, 1H, *J* = 16.4 Hz, -C*H*=C*H*-), 7.82 (d, 1H, *J* = 8.4 Hz, pyridine-*H*), 7.96 (s, 1H, Ar-*H*), 8.13 (d, 1H, *J* = 8.4 Hz, pyridine-*H*), 9.81 (s, br, 1H, O*H*); ^13^C-NMR (100 MHz, DMSO-*d*_6_) *δ* 109.0, 116.3, 122.8, 123.7, 125.3, 127.8, 128.6, 129.1, 131.1, 132.8, 134.2, 148.4, 153.8, 158.8, 164.2; IR (KBr): *ν* 3,436, 2,979, 2,681, 1,635, 1,604, 1,555, 1,515, 1,466, 1,278, 1,247, 1,170 cm^-1^; ESI-MS: *m/z* 340.7 (M^-^-H).

### General Procedure for the Synthesis of (E)-7-Amino-5-chloro-2-styryl-substituted Quinolin-8-ol Derivatives ***6a-d***

To a suspension of (*E*)-5-chloro-7-nitro-2-styryl substituted quinolin-8-ol **5a-d** (5 mmol) in methanol (50 mL) and water (50 mL) was added 40 equivalents of sodium dithionite. The reaction mixture was stirred for 24 hrs at room temperature and then quenched by adding water (100 mL). The formed precipitate was filtered, washed and dried to get desired (*E*)-7-amino-5-chloro-2-styryl substituted quinolin-8-ol derivatives **6a-d.**

*(E)-7-Amino-5-chloro-2-styrylquinolin-8-ol* (**6a**): Yield: 86%; mp: 193–195 ^o^C; ^1^H-NMR (400 MHz, DMSO-*d*_6_) *δ* 5.38 (s, br, 2H, N*H*_2_), 7.23 (s, 1H, Ar-*H*), 7.36 (t, 1H, *J* = 7.2 Hz, Ar-*H*), 7.44 (d, 1H, *J* = 16 Hz, -C*H*=C*H*-), 7.46 (t, 2H, *J* = 7.2 Hz, Ar-*H*), 7.54 (d, 1H, *J* = 8.8 Hz, pPyridine-*H*), 7.72 (d, 2H, *J* = 7.2 Hz, Ar-*H*), 8.15 (d, 1H, *J* = 16.4 Hz, -C*H*=C*H*-), 8.22 (d, 1H, *J* = 8.8 Hz, pyridine-*H*), 9.03 (s, br, 1H, O*H*); IR (KBr): *ν* 3,436, 1,634, 1,508, 1,465, 1,290, 1,193 cm^-1^; ESI-MS: *m/z* 286.9 (M+H)^+^, 294.5 (M^-^-H) ^-^.

*(E)-2-(4-Bromostyryl)-7-amino-5-chloroquinolin-8-ol* (**6b**): Yield: 94%;mp: 201–202 ^o^C; ^1^H-NMR (400 MHz, DMSO-*d*_6_) *δ* 7.59 (d, 1H, *J* = 16 Hz, -C*H*=C*H*-), 7.66 (s, 1H, O*H*), 7.68 (s, 4H, Ar-*H*), 8.08 (d, 1H, *J* = 8.8 Hz, pyridine-*H*), 8.15 (s, 1H, Ar-*H*), 8.34 (d, 1H, *J* = 16.4 Hz, -C*H*=C*H*-), 8.56 (d, 1H, *J* = 8.8 Hz, pyridine-*H*); IR (KBr): *ν* 3,432, 3,080, 1,628, 1,566, 1,516, 1,342, 1,180 cm^-1^; ESI-MS: *m/z* 376.8 (M+H)^+^.

*(E)-2-(4-Methoxystyryl)-7-amino-5-chloroquinolin-8-ol* (**6c**): Yield: 60%; mp: 154–156 ^o^C; ^1^H-NMR (400 MHz, DMSO-*d*_6_) *δ* 3.81 (s, 3H, OC*H*_3_), 5.42 (s br, 2H, N*H*_2_), 7.02 (d, 2H, *J* = 8.4 Hz, Ar-*H*), 7.21 (s, 1H, Ar-*H*), 7.29 (d, 1H, *J* = 16.4 Hz, -C*H*=C*H*-), 7.49 (d, 1H, *J* = 8.4 Hz, pyridine-*H*), 7.66 (d, 2H, *J* = 8.4 Hz, Ar-*H*), 8.08 (d, 1H, *J* = 16 Hz, -C*H*=C*H*-), 8.19 (d, 1H, *J* = 8.8 Hz, pyridine-*H*), 8.99 (s, br, 1H, O*H*); IR (KBr): *ν* 3,450, 3,376, 3,338, 1,606, 1,513, 1,305, 1,176 cm^-1^; ESI-MS: *m/z* 326.9 (M+H)^+^ 324.8 (M^-^-H).

*(E)-2-(4-Hydroxystyryl)-7-amino-5-chloroquinolin-8-ol* (**6d**): Yield: 30%; mp: >300 ^o^C; ^1^H-NMR (400 MHz, DMSO-*d*_6_) *δ* 5.75 (s, br, 2H, N*H*_2_), 6.85 (d, 2H, *J* = 8.4 Hz, Ar-*H*), 7.22 (s, 1H, Ar-*H*), 7.30 (d, 1H, *J* = 16 Hz, -C*H*=C*H*-), 7.54 (d, 1H, *J* = 8.4 Hz, pyridine-*H*), 7.55 (d, 2H, *J* = 8.4 Hz, Ar-*H*), 8.03 (d, 1H, *J* = 16 Hz, -C*H*=C*H*-), 8.23 (d, 1H, *J* = 8.4 Hz, pyridine-*H*), 9.11 (s, br, 1H, O*H*), 9.88 (s, 1H, O*H*); IR (KBr): *ν* 3,413, 3,369, 1,621, 1,514, 1,279, 1,171 cm^-1^; ESI-MS: *m/z* 312.9 (M+H)^+^, 310.7 (M^-^-H)^-^.

### General Procedure for the Synthesis of (E)-N-(2-substituted-styryl)-5-chloro-8-hydroxyquinolin-7-yl)-benzenesulfonamides ***III***

To a flask (50 mL) charged with the appropriate compound **6** (0.5 mmol) and DMAP (0.05 mmol) dissolved in pyridine (10 mL) was added dropwise benzenesulfonic chloride (0.55 mmol) in pyridine (5 mL). After addition, the reaction mixture was stirred for 2 hrs at room temperature and then the solvent was removed under reduced pressure. The residue was dissolved in CH_2_Cl_2_ (50 mL) and washed with water, dried by MgSO_4_. The desired product **III** was obtained after column chromatograph and then recrystallization using petroleum ether and ethyl acetate. 

*(E)-N-(2-(4-Methoxystyryl)-5-chloro-8-hydroxyquinolin-7-yl)-4-methoxybenzenesulfonamide* (**IIIa**): Yield: 44%; mp: 192–193 ^o^C; ^1^H-NMR (400 MHz, DMSO-*d*_6_) *δ* 3.77 (s, 3H, OC*H*_3_), 3.81 (s, 3H, OC*H*_3_), 7.02 (d, 2H, *J* = 8.8 Hz, Ar-*H*), 7.02 (d, 2H, *J* = 8.8 Hz, Ar-*H*), 7.29 (d, 1H, *J* = 16 Hz, -C*H*=C*H*-), 7.56 (s, 1H, Ar-*H*), 7.64 (d, 2H, *J* = 8.8 Hz, Ar-*H*), 7.72 (d, 2H, *J* = 8.8 Hz, Ar-*H*), 7.75 (d, 1H, *J* = 8.8 Hz, pyridine-*H*), 8.23 (d, 1H, *J* = 16 Hz, -C*H*=C*H*-), 8.34 (d, 1H, *J* = 8.8 Hz, pyridine-*H*), 9.91 (s, br, 2H, O*H*, N*H*); ^13^C-NMR (100 MHz, DMSO-*d*_6_) *δ* 55.7, 56.0, 114.7, 114.9, 118.6, 121.2, 122.8, 124.3, 124.9, 129.3, 129.3, 129.3, 129.4, 132.6, 133.4, 136.3, 138.9, 145.4, 155.4, 160.5, 162.8; IR (KBr): *ν* 3,420, 3,293, 2,930, 1,620, 1,593, 1,512, 1,258, 1,158 cm^-1^; ESI-MS: *m/z* 494.7 (M^-^-1), 496.8 (M^+^+1), 518.8 (M^+^+Na).

*(E)-N-(2-(4-Methoxystyryl)-5-chloro-8-hydroxyquinolin-7-yl)-4-methylbenzenesulfonamide* (**IIIb**): Yield: 42%; mp: 202–204 ^o^C; ^1^H-NMR (400 MHz, DMSO-*d*_6_) *δ* 2.32 (s, 3H, C*H*_3_), 3.81 (s, 3H, OC*H*_3_), 7.02 (d, 2H, *J* = 8.8 Hz, Ar-*H*), 7.29 (d, 1H, *J* = 16 Hz, -C*H*=C*H*-), 7.31 (d, 2H, *J* = 8.0 Hz, Ar-*H*), 7.57 (s, 1H, Ar-*H*), 7.64 (d, 2H, *J* = 8.4 Hz, Ar-*H*), 7.69 (d, 2H, *J* = 8.0 Hz, Ar-*H*), 7.75 (d, 1H, *J* = 8.8 Hz, pyridine-*H*), 8.24 (d, 1H, *J* = 16 Hz, -C*H*=C*H*-), 8.34 (d, 1H, *J* = 8.4 Hz, pyridine-*H*), 9.90 (s, br, 1H, O*H*), 10.01 (s, br, 1H, N*H*); ^13^C-NMR (100 MHz, DMSO-*d*_6_) *δ* 21.4, 55.7, 114.9, 118.6, 121.1, 122.4, 122.8, 124.3, 124.9, 127.1, 129.3, 129.4, 130.0, 133.3, 136.3, 138.2, 138.9, 143.5, 145.4, 155.4, 160.5; IR (KBr): *ν* 3,448, 3,290, 1,634, 1,589, 1,513, 1,461, 1,249, 1,158 cm^-1^; ESI-MS: *m/z* 478.8 (M^-^-1), 480.8 (M^+^+1), 502.8 (M^+^+Na).

*(E)-N-(2-(4-Methoxystyryl)-5-chloro-8-hydroxyquinolin-7-yl)-4-chlorobenzenesulfonamide* (**IIIc**): Yield: 49%; mp: 219–221 ^o^C; ^1^H-NMR (400 MHz, DMSO-*d*_6_) δ 3.81 (s, 3H, OC*H*_3_), 7.02 (d, 2H, *J* = 8.8 Hz, Ar-*H*), 7.30 (d, 1H, *J* = 16.4 Hz, -C*H*=C*H*-), 7.54 (s, 1H, Ar-*H*), 7.60 (d, 2H, *J* = 8.8Hz, Ar-*H*), 7.64 (d, 2H, *J* = 8.8 Hz, Ar-*H*), 7.78 (d, 2H, *J* = 8.8 Hz, Ar-*H*), 7.78 (d, 1H, *J* = 8.8 Hz, pyridine-*H*), 8.23 (d, 1H, *J* = 16 Hz, -C*H*=C*H*-), 8.36 (d, 1H, *J* = 8.8 Hz, pyridine-*H*), 9.90 (s br, 1H, O*H*), 10.23 (s br, 1H, N*H*); ^13^C-NMR (100 MHz, DMSO-*d*_6_) *δ* 55.7, 114.9, 118.7, 120.4, 122.5, 123.2, 124.9, 125.2, 129.0, 129.3, 129.4, 129.7, 133.4, 136.4, 138.1, 139.0, 140.0, 146.2, 155.5, 160.5; IR (KBr): *ν* 3,291, 1,632, 1,589, 1,512, 1,464, 1,327, 1,259, 1,161 cm^-1^; ESI-MS: *m/z* 499.7 (M^-^-1), 500.8 (M^+^+1).

*(E)-N-(2-(4-Methoxystyryl)-5-chloro-8-hydroxyquinolin-7-yl)benzenesulfonamide* (**IIId**): Yield: 53%; mp: 237–238 ^o^C; ^1^H-NMR (400 MHz, DMSO-*d*_6_) *δ* 3.80 (s, 3H, OC*H*_3_), 7.00 (d, 2H, *J* = 8.8 Hz, Ar-*H*), 7.27 (d, 1H, *J* = 16 Hz, -C*H*=C*H*-), 7.51 (t, 2H, *J* = 7.2 Hz, Ar-*H*), 7.55 (s, 1H, Ar-*H*), 7.57 (t, 1H, *J* = 7.2 Hz, Ar-*H*), 7.62 (d, 2H, *J* = 8.8 Hz, Ar-*H*), 7.73 (d, 1H, *J* = 8.8 Hz, pyridine-*H*), 7.81 (d, 2H, *J* = 8.8 Hz, Ar-*H*), 8.22 (d, 1H, *J* = 16 Hz, -C*H*=C*H*-), 8.31 (d, 1H, *J* = 8.8 Hz, pyridine-*H*), 10.10 (s, br, 2H, O*H*, N*H*); ^13^C-NMR (100 MHz, DMSO-*d*_6_) *δ* 55.7, 114.8, 118.7, 120.9, 122.3, 122.9, 124.5, 124.9, 127.0, 129.3, 129.4, 129.5, 133.2, 133.3, 136.3, 138.9, 141.0, 145.7, 155.5, 160.5; IR (KBr): *ν* 3,371, 3,241, 1,627, 1,513, 1,455, 1,329, 1,249, 1,172 cm^-1^; ESI-MS: *m/z* 464.8 (M^—^1), 466.8 (M^+^+1), 488.8 (M^+^+Na); Anal. Calcd for C_24_H_19_ClN_2_O_4_S: C 61.73, H 4.10, N 6.00; found C 61.48, H 3.98, N 6.00. 

*(E)-N-(5-Chloro-8-hydroxy-2-styrylquinolin-7-yl)-4-chlorobenzenesulfonamide* (**IIIe**): Yield: 52%; mp: 159–160 ^o^C; ^1^H-NMR (400 MHz, DMSO-*d*_6_) *δ* 7.36 (t, 1H, *J* = 7.2 Hz, Ar-*H*), 7.45 (t, 2H, *J* = 7.2 Hz, Ar-*H*), 7.45 (t, 1H, *J* = 16 Hz, -C*H*=C*H*-), 7.56 (s, 1H, Ar-*H*), 7.59 (d, 2H, *J* = 7.2Hz, Ar-*H*), 7.69 (d, 2H, *J* = 7.6 Hz, Ar-*H*), 7.78 (d, 2H, *J* = 7.2Hz, Ar-*H*), 7.82 (d, 1H, *J* = 8.8 Hz, pyridine-*H*), 8.28 (d, 1H, *J* = 16.4 Hz, -C*H*=C*H*-), 8.38 (d, 1H, *J* = 8.8 Hz, pyridine-*H*), 10.00 (s, br, 1H, O*H*), 10.26 (s, br, 1H, N*H*); ^13^C-NMR (100 MHz, DMSO-*d*_6_) *δ* 118.7, 120.5, 122.7, 123.4, 125.5, 127.3, 127.8, 129.0, 129.4, 129.7, 133.5, 136.6, 136.7, 138.1, 139.0, 140.0, 146.3, 155.1; IR (KBr): *ν* 3,430, 3,299, 1,633, 1,587, 1,465, 1,328, 1,094 cm^-1^; ESI-MS: *m/z* 468.8 (M^-^-1), 470.8 (M^+^+1). 

*(E)-N-(5-Chloro-8-hydroxy-2-styrylquinolin-7-yl)-4-methylbenzenesulfonamide* (**IIIf**): Yield: 51%; mp: 186–188 ^o^C; ^1^H-NMR (400 MHz, DMSO-*d*_6_) *δ* 2.32 (s, 3H, C*H*_3_), 7.31 (d, 2H, *J* = 8.0 Hz, Ar-*H*), 7.37 (t, 1H, *J* = 7.2 Hz, Ar-*H*), 7.45 (t, 2H, *J* = 7.2 Hz, Ar-*H*), 7.45 (d, 1H, *J* = 15.6 Hz, -C*H*=C*H*-), 7.59 (s, 1H, Ar-*H*), 7.68 (d, 2H, *J* = 8.2 Hz, Ar-*H*), 7.69 (d, 2H, *J* = 7.2 Hz, Ar-*H*), 7.79 (d, 1H, *J* = 8.8 Hz, pyridine-*H*), 8.30 (d, 1H, *J* = 16 Hz, -C*H*=C*H*-), 8.38 (d, 1H, *J* = 8.4 Hz, pyridine-*H*), 10.03 (s, br, 2H, O*H*, N*H*); ^13^C-NMR (100 MHz, DMSO-*d*_6_) *δ* 21.4, 118.6, 121.1, 122.6, 123.0, 124.6, 127.1, 127.3, 127.8, 129.4, 130.0, 133.5, 136.5, 136.7, 138.2, 138.9, 143.5, 145.6, 155.0; IR (KBr): *ν* 3,437, 3,295, 1,633, 1,592, 1,462, 1,322, 1,158, 1089 cm^-1^; ESI-MS: *m/z* 448.7 (M^-^-1), 450.9 (M^+^+1). 

*(E)-N-(5-Chloro-8-hydroxy-2-styrylquinolin-7-yl)-4-methoxybenzenesulfonamide* (**IIIg**): Yield: 54%; mp: 193–195 ^o^C; ^1^H-NMR (400 MHz, DMSO-*d*_6_) *δ* 3.77 (s, 3H, OC*H*_3_), 7.03 (d, 2H, *J* = 8.8 Hz, Ar-*H*), 7.37 (t, 1H, *J* = 7.2 Hz, Ar-*H*), 7.45 (t, 2H, *J* = 8.0 Hz, Ar-*H*), 7.45 (d, 1H, *J* = 16 Hz, -C*H*=C*H*-), 7.58 (s, 1H, Ar-*H*), 7.69 (d, 2H, *J* = 7.6 Hz, Ar-*H*), 7.72 (d, 2H, *J* = 8.8 Hz, Ar-*H*), 7.80 (d, 1H, *J* = 8.8 Hz, pyridine-*H*), 8.29 (d, 1H, *J* = 16 Hz, -C*H*=C*H*-), 8.38 (d, 1H, *J* = 8.4Hz, pyridine-*H*), 9.92 (s, br, 2H, O*H*, N*H*); ^13^C-NMR (100 MHz, DMSO-*d*_6_) *δ* 56.0, 114.7, 118.6, 121.3, 122.5, 123.0, 124.6, 127.3, 127.8, 129.3, 129.38, 132.6, 133.5, 136.5, 136.8, 138.9, 145.5, 155.0, 162.8; IR (KBr): *ν* 3,376, 3,236, 1,627, 1,595, 1,499, 1,459, 1,262, 1,155, 1,092 cm^-1^; ESI-MS: *m/z* 464.7 (M^-^-1), 466.9 (M^+^+1), 488.9 (M^+^+Na); Anal. Calcd for C_24_H_19_ClN_2_O_4_S: C 61.73, H 4.10, N 6.00; found C 61.67, H 4.14, N 6.02.

*(E)-N-(5-Chloro-8-hydroxy-2-styrylquinolin-7-yl)benzenesulfonamide* (**IIIh**): Yield: 46%; mp: 200–201 ^o^C; ^1^H-NMR (400 MHz, DMSO-*d*_6_) *δ* 7.37 (t, 1H, *J* = 7.2 Hz, Ar-*H*), 7.45 (d, 1H, *J* = 16 Hz -C*H*=C*H*-), 7.45 (t, 2H, *J* = 7.6 Hz, Ar-*H*), 7.52 (t, 2H, *J* = 7.2 Hz, Ar-*H*), 7.57 (s, 1H, Ar-*H*), 7.60 (t, 1H, *J* = 7.2 Hz, Ar-*H*), 7.70 (d, 2H, *J* = 7.2 Hz, Ar-*H*), 7.81 (d, 2H, *J* = 7.2 Hz, Ar-*H*), 7.81 (d, 1H, *J* = 8.8 Hz, pyridine-*H*), 8.29 (d, 1H, *J* = 16 Hz, -C*H*=C*H*-), 8.38 (d, 1H, *J* = 8.8 Hz, pyridine-*H*), 9.98 (s, br, 1H, O*H*), 10.08 (s, br, 1H, N*H*); ^13^C-NMR (100 MHz, DMSO-*d*_6_) *δ* 118.7, 121.0, 122.5, 123.1, 124.8, 127.0, 127.3, 127.8, 129.4, 129.6, 133.3, 133.5, 136.5, 136.7, 138.9, 141.0, 145.8, 155.1; IR (KBr): *ν* 3,304, 1,633, 1,589, 1,506, 1,314, 1,160, 1,090 cm^-1^; ESI-MS: *m/z* 434.7 (M^-^-1), 436.9 (M^+^+1).

*(E)-N-(5-Chloro-8-hydroxy-2-styrylquinolin-7-yl)-4-nitrobenzenesulfonamide* (**IIIi**): Yield: 56%; mp: 247–249 ^o^C; ^1^H-NMR (400 MHz, DMSO-*d*_6_) *δ* 7.36 (t, 1H, *J* = 7.2 Hz, Ar-*H*), 7.45 (t, 2H, *J* = 8.0 Hz, Ar-*H*), 7.45 (d, 1H, *J* = 16 Hz, -C*H*=C*H*-), 7.58 (s, 1H, Ar-*H*), 7.68 (d, 2H, *J* = 7.2 Hz, Ar-*H*), 7.85 (d, 1H, *J* = 8.8 Hz, pyridine-*H*), 8.03 (dt, 2H, *J* = 8.8 Hz, *J* = 2.4 Hz, Ar-*H*), 8.25 (d, 1H, *J* = 16 Hz, -C*H*=C*H*-), 8.35 (dt, 2H, *J* = 8.8 Hz, *J* = 2.4 Hz, Ar-*H*), 8.41 (d, 1H, *J* = 8.8 Hz, pyridine-*H*), 9.98 (s, br, 1H, O*H*), 10.53 (s, br, 1H, N*H*); ^13^C-NMR (100 MHz, DMSO-*d*_6_) *δ* 118.8, 120.0, 122.8, 123.6, 124.9, 125.9, 127.3, 127.8, 128.7, 129.4, 133.5, 136.6, 136.7, 139.0, 146.7, 146.8, 150.2, 155.2; IR (KBr): *ν* 3,388, 3,255, 1,519, 1,347, 1,313, 1,172 cm^-1^; ESI-MS: *m/z* 479.6 (M^-^-1), 503.9 (M^+^+Na); Anal. Calcd for C_23_H_16_ClN_3_O_5_S: C 57.32, H 3.35, N 8.72; found C 57.56, H 3.46, N 8.66.

*(E)-N-(2-(4-Bromostyryl)-5-chloro-8-hydroxyquinolin-7-yl)-4-methoxybenzenesulfonamide* (**IIIj**): Yield; 25%; mp: 206–208 ^o^C; ^1^H-NMR (400 MHz, DMSO-*d*_6_) *δ* 3.76 (s, 3H, OC*H*_3_), 7.02 (dt, 2H, *J* = 8.8 Hz, *J* = 2.4 Hz, Ar-*H*), 7.47 (d, 1H, *J* = 16 Hz, -C*H*=C*H*-), 7.60 (s, 1H, Ar-*H*), 7.62 (s, 4H, Ar-*H*), 7.72 (dt, 2H, *J* = 8.8 Hz, *J* = 2.4 Hz, Ar-*H*), 7.76 (d, 1H, *J* = 8.8 Hz, pyridine-*H*), 8.26 (d, 1H, *J* = 16 Hz, -C*H*=C*H*-), 8.36 (d, 1H, *J* = 8.8 Hz, pyridine-*H*), 9.94 (s, br, 2H, O*H*, N*H*); ^13^C-NMR (100 MHz, DMSO-*d*_6_) *δ* 56.0, 114.7, 118.7, 121.3, 122.4, 122.6, 123.0, 124.7, 128.1, 129.3, 129.7, 132.3, 132.6, 122.6, 135.1, 136.0, 138.9, 145.5, 154.7, 162.8; IR (KBr): *ν* 3,291, 1,594, 1,463, 1,261, 1,157, 1,090 cm^-1^; ESI-MS: *m/z* 544.7 (M^-^-1); Anal. Calcd for C_24_H_18_BrClN_2_O_4_S: C 52.81, H 3.32, N 5.13; found C 52.73, H 3.45, N 5.11.

*(E)-N-(2-(4-Bromostyryl)-5-chloro-8-hydroxyquinolin-7-yl)-4-methylbenzenesulfonamide* (**IIIk**): Yield: 51%; mp: 234–236 ^o^C; ^1^H-NMR (400 MHz, DMSO-*d*_6_) *δ* 2.32 (s, 3H, C*H*_3_), 7.31 (d, 2H, *J* = 8.0 Hz, Ar-*H*), 7.48 (d, 1H, *J* = 16 Hz, -C*H*=C*H*-), 7.59 (s, 1H, Ar-*H*), 7.63-7.64 (m, 4H, Ar-*H*), 7.68 (d, 2H, *J* = 8.0 Hz, Ar-*H*), 7.78 (d, 1H, *J* = 8.8 Hz, pyridine-*H*), 8.29 (d, 1H, *J* = 16 Hz, -C*H*=C*H*-), 8.38 (d, 1H, *J* = 8.4 Hz, pyridine-*H*), 9.98 (s, br, 1H, O*H*), 10.04 (s, br, 1H, N*H*); ^13^C-NMR (100 MHz, DMSO-*d*_6_) *δ* 21.4, 118.6, 121.2, 122.4, 122.7, 123.1, 124.8, 127.1, 128.1, 129.7, 130.0, 132.4, 133.6, 135.1, 136.1, 138.2, 138.9, 143.5, 145.5, 154.7; IR (KBr): *ν* 3,291, 1,634, 1,585, 1,460, 1,310, 1,158 cm^-1^; ESI-MS: *m/z* 528.6 (M^-^-1).

*(E)-N-(2-(4-Bromostyryl)-5-chloro-8-hydroxyquinolin-7-yl)benzenesulfonamide* (**IIIl**): Yield: 38%; mp: 238–239 ^o^C; ^1^H-NMR (400 MHz, DMSO-*d*_6_) *δ* 7.48 (d, 1H, *J* = 16 Hz, -C*H*=C*H*-), 7.52 (t, 2H, *J* = 7.2 Hz, Ar-*H*), 7.58 (s, 1H, Ar-*H*), 7.60 (t, 1H, *J* = 8.4 Hz, Ar-*H*), 7.64 (s, 4H,, Ar-*H*), 7.65 (d, 2H, *J* = 9.6Hz, Ar-*H*), 7.78 (d, 1H, *J* = 8.8 Hz, pyridine-*H*), 7.80 (d, 2H, *J* = 7.2 Hz, Ar-*H*), 8.28 (d, 1H, *J* = 16 Hz, -C*H*=C*H*-), 8.39 (d, 1H, *J* = 8.8 Hz, pyridine-*H*), 9.98 (s, br, 1H, O*H*), 10.14 (s, br, 1H, N*H*); ^13^C-NMR (100 MHz, DMSO-*d*_6_) *δ* 118.6, 121.0, 122.4, 122.7, 123.2, 124.9, 127.0, 128.1, 129.5, 129.7, 132.4, 133.3, 133.6, 135.2, 136.0, 138.9, 141.0, 145.8, 154.7; IR (KBr): *ν* 2,924, 2,853, 1,459, 1,310, 1,159 cm^-1^; ESI-MS: *m/z* 514.5 (M^-^-1), 538.9(M^+^+Na).

*(E)-N-(2-(4-Bromostyryl)-5-chloro-8-hydroxyquinolin-7-yl)-4-chlorobenzenesulfonamide* (**IIIm**): Yield: 35%; mp: 238–240 ^o^C; ^1^H-NMR (400 MHz, DMSO-*d*_6_) *δ* 7.44 (d, 1H, *J* = 16 Hz, -C*H*=C*H*-), 7.57 (s, 1H, Ar-*H*), 7.60 (d, 2H, *J* = 7.2 Hz, Ar-*H*), 7.61 (s, 4H, Ar-*H*), 7.76 (d, 1H, *J* = 8.8 Hz, pyridine-*H*), 7.79 (d, 2H, *J* = 7.2 Hz, Ar-*H*), 8.24 (d, 1H, *J* = 16 Hz, -C*H*=C*H*-), 8.35 (d, 1H, *J* = 8.8 Hz, pyridine-*H*), 10.15 (s, br, 2H, O*H*, N*H*); ^13^C-NMR (100 MHz, DMSO-*d*_6_) *δ* 118.7, 120.5, 122.4, 122.7, 123.4, 125.6, 128.1, 129.0, 129.7, 132.3, 133.6, 135.2, 136.0, 138.1, 138.9, 139.9, 146.3, 154.8; IR (KBr): *ν* 3,435, 3,292, 1,633, 1,461, 1,326, 1,161 cm^-1^; ESI-MS: *m/z* 548.5 (M^-^-1), 572.9(M^+^+Na).

*(E)-N-(2-(4-Hydroxystyryl)-5-chloro-8-hydroxyquinolin-7-yl)-4-methoxybenzenesulfonamide* (**IIIn**): Yield: 33%; mp: 212–213 ^o^C; ^1^H-NMR (400 MHz, DMSO-*d*_6_) *δ* 3.76 (s, 3H, OC*H*_3_), 6.84 (d, 2H, *J* = 8.8 Hz, Ar-*H*), 7.01 (dt, 2H, *J* =7.2 Hz, *J* = 2.4 Hz, Ar-*H*), 7.21 (d, 1H, *J* = 16 Hz, -C*H*=C*H*-), 7.53 (d, 2H, *J* = 8.4 Hz, Ar-*H*), 7.55 (s, 1H, Ar-*H*), 7.72 (dt, 2H, *J* = 7.2Hz, *J* = 2.4 Hz, Ar-*H*), 7.73 (d, 1H, *J* = 8.8 Hz, pyridine-*H*), 8.17 (d, 1H, *J* = 16.4 Hz, -C*H*=C*H*-), 8.31 (d, 1H, *J* = 8.8 Hz, pyridine-*H*), 9.80-9.95 (m, br, 3H, O*H*, N*H*); ^13^C-NMR (100 MHz, DMSO-*d*_6_) *δ* 56.0, 114.7, 116.3, 118.6, 121.2, 122.2, 122.7, 124.0, 124.2, 127.8, 129.3, 129.5, 132.6, 133.2, 136.7, 138.9, 145.3, 155.6, 159.0, 162.8; IR (KBr): *ν* 3,430, 3,251, 1,630, 1,590, 1,498, 1,438, 1,319, 1,264, 1,147 cm^-1^; ESI-MS: *m/z* 480.7 (M^-^-1), 483.0 (M^+^+1), 505.0 (M^+^+Na); Anal. Calcd for C_24_H_19_ClN_2_O_5_S: C 59.69, H 3.97, N 5.80; found C 59.41, H 4.05, N 5.64.

*(E)-N-(2-(4-Hydroxystyryl)-5-chloro-8-hydroxyquinolin-7-yl)-4-methylbenzenesulfonamide* (**IIIo**): Yield: 31%; mp: 214–215 ^o^C; ^1^H-NMR (400 MHz, DMSO-*d*_6_) *δ* 2.32 (s, 3H, C*H*_3_), 6.84 (d, 2H, *J* = 8.4 Hz, Ar-*H*), 7.21 (d, 1H, *J* = 16 Hz, -C*H*=C*H*-), 7.31 (d, 2H, *J* = 8.0 Hz, Ar-*H*), 7.53 (d, 2H, *J* = 8.8 Hz, Ar-*H*), 7.55 (s, 1H, Ar-*H*), 7.68 (d, 2H, *J* = 8.0 Hz, Ar-*H*), 7.73 (d, 1H, *J* = 8.8 Hz, pyridine-*H*), 8.19 (d, 1H, *J* = 16 Hz, -C*H*=C*H*-), 8.32 (d, 1H, *J* = 8.4 Hz, pyridine-*H*), 9.83 (s, 1H, O*H*), 9.89 (s, 1H, O*H*), 10.02 (s, 1H, N*H*); ^13^C-NMR (100 MHz, DMSO-*d*_6_) *δ* 21.4, 116.3, 118.6, 121.0, 122.3, 122.7, 123.9, 124.2, 127.1, 127.8, 129.5, 130.0, 133.3, 136.8, 138.2, 138.9, 143.5, 145.4, 155.6, 159.0; IR (KBr): *ν* 3,434, 3,258, 1,589, 1,515, 1,458, 1,279, 1,185, 1,150 cm^-1^; ESI-MS: *m/z* 464.8 (M^-^-1), 466.9 (M^+^+1), 488.9 (M^+^+Na); Anal. Calcd for C_24_H_19_ClN_2_O_4_S: C 61.73, H 4.10, N 6.00.

*(E)-N-[2-(4-Hydroxystyryl)-5-chloro-8-hydroxyquinolin-7-yl]benzenesulfonamide* (**IIIp**): Yield: 25%; mp: 191–193 ^o^C; ^1^H-NMR (400 MHz, DMSO-*d*_6_) *δ* 6.84 (d, 2H, *J* = 8.8 Hz, Ar-*H*), 7.22 (d, 1H, *J* = 16 Hz, -C*H*=C*H*-), 7.50-7.54 (m, 5H, Ar-*H*), 7.60 (tt, 1H, *J* = 6.8 Hz, *J* = 2.0 Hz, Ar-*H*), 7.74 (d, 1H, *J* = 8.8 Hz, pyridine-*H*), 7.79 (dt, 2H, *J* = 7.2 Hz, *J* = 2.0 Hz, Ar-*H*), 8.19 (d, 1H, *J* = 16 Hz, -C*H*=C*H*-), 8.33 (d, 1H, *J* = 8.8 Hz, pyridine-*H*), 9.86 (s, 1H, O*H*), 9.91 (s, 1H, O*H*), 10.15 (s, 1H, N*H*); ^13^C- NMR (100 MHz, DMSO-*d*_6_) *δ* 116.3, 118.6, 120.8, 122.3, 122.8, 1238, 124.4, 127.0, 127.8, 129.5, 129.6, 133.3, 136.8, 138.8, 140.9, 145.6, 155.6, 159.0; IR (KBr): *ν* 3,392, 1,625,1,585, 1,513, 1,455, 1,337, 1,154, 1,091 cm^-1^; ESI-MS: *m/z* 450.7 (M^-^-1), 475.0 (M^+^+Na).

*(E)-N-(2-(4-Hydroxystyryl)-5-chloro-8-hydroxyquinolin-7-yl)-4-chlorobenzenesulfonamide* (**IIIq**): Yield: 16%; mp: 189–191 ^o^C; ^1^H-NMR (400 MHz, acetone-*d*_6_) *δ* 6.91 (d, 2H, *J* = 8.4 Hz, Ar-*H*), 7.24 (d, 1H, *J* = 16 Hz, -C*H*=C*H*-), 7.53 (d, 2H, *J* = 8.4 Hz, Ar-*H*), 7.57 (d, 2H, *J* = 8.8 Hz, Ar-*H*), 7.77 (s, 1H, Ar-*H*), 7.79 (d, 1H, *J* = 8.8 Hz, pyridine-*H*), 7.86 (d, 2H, *J* = 8.4 Hz, Ar-*H*), 8.05 (d, 1H, *J* = 16 Hz, -C*H*=C*H*-), 8.40 (d, 1H, *J* = 8.8 Hz, pyridine-*H*), 8.82 (s, br, 3H, O*H*, O*H*, N*H*); ^13^C-NMR (100 MHz, acetone-*d*_6_) *δ* 115.8, 119.6, 120.2, 121.6, 122.8, 123.4, 123.8, 128.1, 128.9, 129.1, 129.2, 133.1, 136.73, 136.2, 138.4, 138.5, 144.0, 155.8, 158.6; IR (KBr): *ν* 3,252, 1,630, 1,514, 1,274, 1,168, 1,091 cm^-1^; ESI-MS: *m/z* 484.8 (M^-^-1), 486.9 (M^+^+1), 508.9 (M^+^+Na).

### HIV-IN inhibitory activity evaluation

Compounds **III** (5 × 10^-5^ mmol) diluted in DMSO (1 mL) were pre-incubated with 800 ng IN at 37 °C in the reaction buffer in the absence of Mn^2+^ for 10 min. Subsequently, 1.5 pmol donor DNA and 9 pmol target DNA were added and the reaction was initiated by the addition of Mn^2+^ (10 mmol/L) into the final reaction volume. The reactions were carried out at 37 ^o^C for 1 h and subsequent detection procedure was applied to detect the assay signals. In these experiments, baicalein, a known IN inhibitor with both viral replication inhibitory effect *in vivo* and IN reaction activities inhibitory effect *in vitro*, was used as the control compound (positive control), whereas 10% DMSO solution without sample was set as the drug-free control (negative control).

## Conclusions

In summary, on the basis of our previous CoMFA analysis of styrylquinoline derivatives, we have designed and synthesized for the first time a series of *N*-[(2-substituted-styryl)-5-chloro-8-hydroxyquinolin-7-yl]-benzenesulfonamide derivatives. The structures of these compounds were characterized and their HIV IN inhibitory activities were evaluated. Results indicate that most of the title compounds exhibit moderate inhibitory activity. Improved inhibitory activity can be achieved when free hydroxyl at the styryl moiety and nitro group at the benzenesulfonamide moiety are present.

## Figures and Tables

**Table 1:**
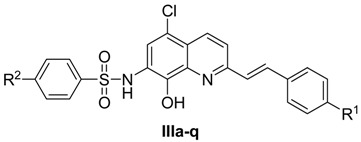
The yields of styrylquinolin-7-yl-benzenesulfonamide derivatives **III**.

No.	R^1^	R^2^	Yield	No.	R^1^	R^2^	Yield
**IIIa**	OCH_3_	OCH_3_	45%	**IIIj**	Br	OCH_3_	25%
**IIIb**	OCH_3_	CH_3_	42%	**IIIk**	Br	CH_3_	51%
**IIIc**	OCH_3_	Cl	49%	**IIIl**	Br	H	38%
**IIId**	OCH_3_	H	53%	**IIIm**	Br	Cl	35%
**IIIe**	H	Cl	52%	**IIIn**	OH	OCH_3_	33%
**IIIf**	H	CH_3_	51%	**IIIo**	OH	CH_3_	31%
**IIIg**	H	OCH_3_	54%	**IIIp**	OH	H	25%
**IIIh**	H	H	46%	**IIIq**	OH	Cl	16%
**IIIi**	H	NO_2_	56%				

**Table 2:** Inhibitory rate of synthesized styrylquinolin-7-yl-benzenesulfonamide derivatives **III**.

Compound	Inhibitory rate % (0.05 mmol/L)	Compound	Inhibitory rate % (0.05 mmol/L)
Baicalein (positive control)	100 (IC_50_ = 1.06 μM)^a^	Negative control (10% DMSO)	0
FZ41	(IC_50_ = 0.7 μM)^b^	**IIIi**	96.7
**IIIa**	11.8	**IIIj**	53.6
**IIIb**	54.6	**IIIk**	59.5
**IIIc**	58.2	**IIIl**	74.7
**IIId**	60.3	**IIIm**	67.8
**IIIe**	82.7	**IIIn**	101.0
**IIIf**	82.0	**IIIo**	100.6
**IIIg**	72.9	**IIIp**	101.4
**IIIh**	95.9	**IIIq**	101.4

^a^ Value of IC_50_ cited from reference [14]; ^b^ Value of IC_50_ cited from reference [6].

## References

[1] Debyser Z., Cherepanov P., Maele B.V., De Clercq E., Witvrouw M. (2002). In search of authentic inhibitors of HIV-1 integration. Antivir. Chem. Chemother..

[2] De Clercq E. (2002). Strategies in the design of antiviral drugs. Nat. Rev. Drug Disc..

[3] Reader J.C. (2004). Automation in medicinal chemistry. Curr. Top. Med. Chem..

[4] Neamati N. (2002). Patented small molecule inhibitors of HIV-1 integrase: A 10-year saga. Exp. Opin. Ther. Pat..

[5] Mekouar K., Mouscadet J.F., Desmaele D., Subra F., Leh H., Savoure D., Auclair C., d’Angelo J. (1998). Styrylquinoline derivatives: A new class of potent HIV-1 integrase inhibitors that block HIV-1 replication in CEM cells. J. Med. Chem..

[6] Zouhiri F., Mouscadet J.F., Mekouar K., Desmaele D., Savoure D., Leh H., Subra F., Bret M.L., Auclair C., d’Angelo J. (2000). Structure-activity relationships and binding mode of styrylquinolines as potent inhibitors of HIV-1 integrase and replication of HIV-1 in cell culture. J. Med. Chem..

[7] Polanski J., Zouhiri F., Jeanson L., Desmaele D., d’Angelo J., Mouscadet J.-F., Gieleciak R., Gasteiger J., Bret L.M. (2002). Use of the Kohonen neural network for rapid screening of *ex vivo* anti-HIV activity of styrylquinolines. J. Med. Chem..

[8] Normand-Baylea M., Bénarda C., Zouhiria F., Mouscadetc J.F., Lehb H., Thomasb C.M., Mbembac G., Desmaëlea D., d’Angeloa J. (2005). New HIV-1 replication inhibitors of the styrylquinoline class bearing aroyl/acyl groups at the C-7 position: Synthesis and biological activity. Bioorg. Med. Chem. Lett..

[9] Ma X.H., Zhang X.Y., Tan J.J., Chen W.Z., Wang C.X. (2004). Exploring binding mode for styrylquinoline HIV-1 integrase inhibitors using comparative molecular field analysis and docking studies. Acta Pharmacol. Sin..

[10] Zeng C.C., Niu L.T., Ping D.W., Zhong R.G. (2009). Design and synthesis of 2-styrylquinoline-7-sulfonamide derivatives as potential HIV integrase inhibitors (in Chinese). Chin. J. Org. Chem..

[11] Yoo H., Lee J.Y., Park J.H., Chung B.Y., Lee Y.S. (2003). Synthesis of styrylbenzofuran derivatives as styrylquinoline analogues for HIV-1 integrase inhibitors. Farmaco.

[12] Kurita K. (1974). Selectivity in tosylation of *o*-aminophenol by choice of tertiary amine. Chem. Ind..

[13] Sellarajah S., Lekishvili T., Bowring C., Thompsett A.R., Rudyk H., Birkett C.R., Brown D.R., Gilbert I.H. (2004). Synthesis of analogues of congo red and evaluation of their anti-prion activity. J. Med. Chem..

[14] He H.Q., Ma X.H., Liu B., Zhang X.Y., Chen W.Z., Wang C.X., Cheng S.H. (2008). A novel high-throughput format assay for HIV-1 integrase strand transfer reaction using magnetic beads. Acta Pharmacol. Sin..

[15] Zouhiri F., Danet M., Bénard C., Normand-Bayle M., Mouscadet J.F., Leh H., Thomas C.M., Mbemba G., d’Angelo J., Desmaële D. (2005). Tetrahedron Lett..

[16] Weizmann M., Bograchov E. (1947). Derivatives of 5-chloro-8-hydroxyquinoline. J. Am. Chem. Soc..

